# Exercise-Induced Cardiac Remodeling: Lessons from Humans, Horses, and Dogs

**DOI:** 10.3390/vetsci4010009

**Published:** 2017-02-12

**Authors:** Rob Shave, Glyn Howatson, Dave Dickson, Lesley Young

**Affiliations:** 1 Cardiff Centre for Exercise and Health, Cardiff Metropolitan University, Cardiff CF23 6XD, UK; 2 Faculty of Health and Life Sciences, Northumbria University, Newcastle upon Tyne TN1 8ST, UK; glyn.howatson@northumbria.ac.uk; 3 Water Research Group, North-West University, Potchefstroom 2520, South Africa; 4 Heart Vets, Porthcawl, Wales CF36 5LD, UK; dave@heartvets.co.uk; 5 Specialist equine Cardiology Services, Moulton, Suffolk CB8 8SD, UK; lesleyeyoung@gmail.com

**Keywords:** echocardiography, exercise physiology, cardiac remodelling

## Abstract

Physical activity is dependent upon the cardiovascular system adequately delivering blood to meet the metabolic and thermoregulatory demands of exercise. Animals who regularly exercise therefore require a well-adapted heart to support this delivery. The purpose of this review is to examine cardiac structure, and the potential for exercise-induced cardiac remodeling, in animals that regularly engage in strenuous activity. Specifically, we draw upon the literature that has studied the “athlete’s heart” in humans, horses, and dogs, to enable the reader to compare and contrast cardiac remodeling in these three athletic species. The available literature provides compelling evidence for exercise-induced cardiac remodeling in all three species. However, more work is required to understand the influence of species/breed specific genetics and exercise-related hemodynamics, in order to fully understand the impact of exercise on cardiac structure.

## 1. Introduction

**Figure 1:**
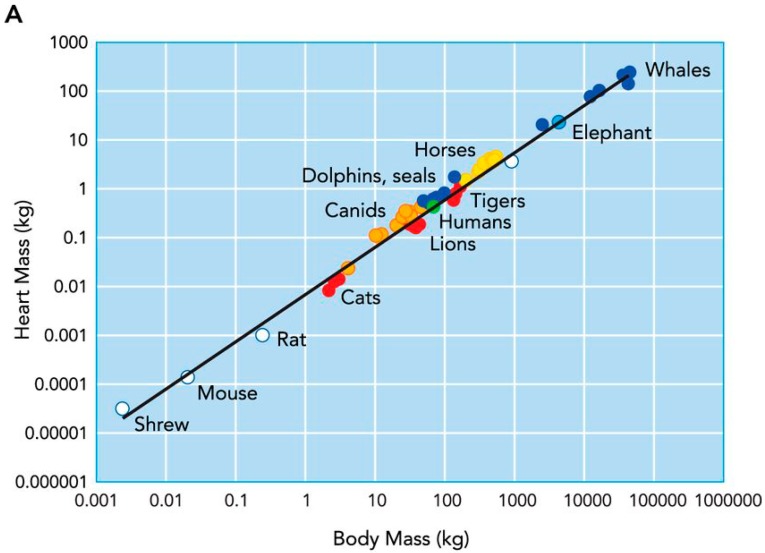
Relationship between heart mass and body mass across the mammalian species. Taken with permission from Williams et al. [1].

**Figure 2:**
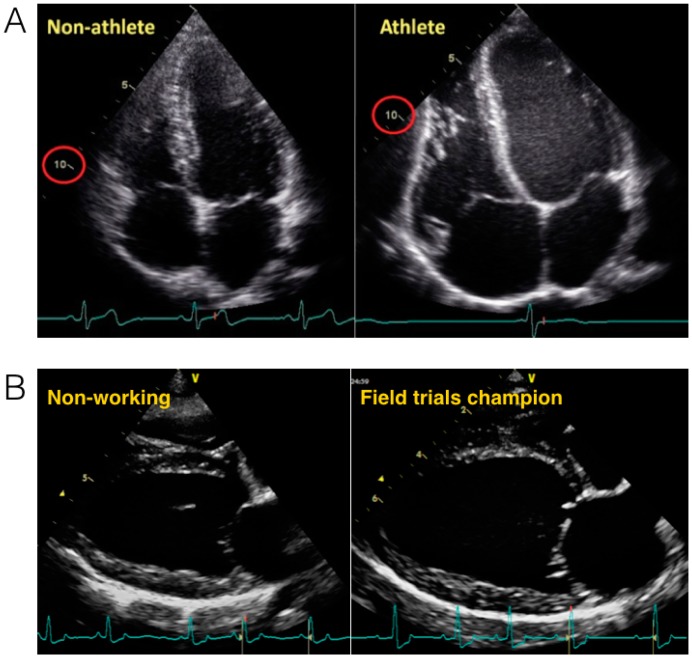
(**A**) Morphological changes in an athlete’s heart compared with a non-athlete’s heart. The normal heart of a 24-year-old librarian (**left**) is compared with that of a 23-year-old professional cyclist (**right**). Note the difference in scale such that the non-athlete’s ventricle is ~10 cm in length (circled in red), whereas the 10-cm mark reaches only halfway along the ventricle of the athlete. Note that all cardiac chambers are enlarged and that increases in wall thickness are relative to dilation. Taken with permission from La Gerche et al. [4]; (**B**) Morphological changes in a working dog’s (English Springer Spaniel) heart. The non-working dog (66 months old, 19 kg) (**left**) is compared with that of a Field trials champion (58 months old, 15.3 kg) (**right**). End diastolic volume in the non-working dog is 47 mL and 58 mL in the field trials champion.

Across mammalian species, heart size increases with body size [1]; proportionate with an increased metabolic demand and the requirement to deliver a large blood volume. Early work from Stahl [2] showed that a mammal’s heart size typically represents ~0.6% of their body mass (Figure 1). However, a closer inspection of individual mammalian species shows that there is considerable variance, and that in some animals, heart size represents as much as 1.7% of their body mass [3]. This variability appears to be related to the type of physical activity that the animals habitually engage in. Specifically, animals that run for prolonged periods of time, either for hunting (e.g., wolves) or sports performance (e.g., Thoroughbred horses and Greyhounds), have markedly larger hearts than those who are less physically active [1] (Figure 2). This exercise-induced cardiac remodeling is also clearly observed in human endurance athletes (Figure 2) [4], and has been termed the athlete’s heart.

Remodeling of the heart in both humans and animals often reflects a maladaptation in response to disease or underlying pathology. Enlargement of the ventricular cavities, or increases in myocardial wall thickness, occur in response to an enhanced volume load or an increase in wall stress, respectively. Thus, the altered hemodynamics associated with cardiac disease, such as valvular regurgitation or aortic stenosis, result in adverse pathologic cardiac remodeling. However, physiologic changes to hemodynamic loading of the heart are also unavoidable during exercise. Dependent on the specific exercise stimulus, the heart may acutely experience an increased volume load, an enhanced “pressure” afterload, or indeed a combination of both. Therefore, when exercise is regularly repeated over a prolonged period of time in a structured manner, akin to that prescribed to competitive athletes (human or animal), there are clear stimuli for cardiac adaptation and remodeling.

The purpose of this review is to examine the physiologic adaptations associated with the athlete’s heart, drawing upon human, canine, and equine literature. Although there is crossover, and the potential for clinical confusion between pathologic and physiologic cardiac remodeling [5,6] this topic will not be addressed here. For more information regarding the differentiation of pathophysiologic remodeling, or on the cardiac pathology responsible for sudden cardiac death in sport, readers are directed to previous reviews [7,8,9,10,11]. Remodeling in human athletes is well recognized; however, in animal athletes the concept has received less attention. Accordingly, the present review will initially discus the characteristics of the human athlete’s heart and the stimuli responsible for athletic remodeling. We will then examine the current evidence for exercise-induced cardiac remodeling in domesticated animal athletes, namely racehorses and dogs involved in athletic pursuits.

### 1.1. Acute Exercise and Cardiac Loading

**Figure 3:**
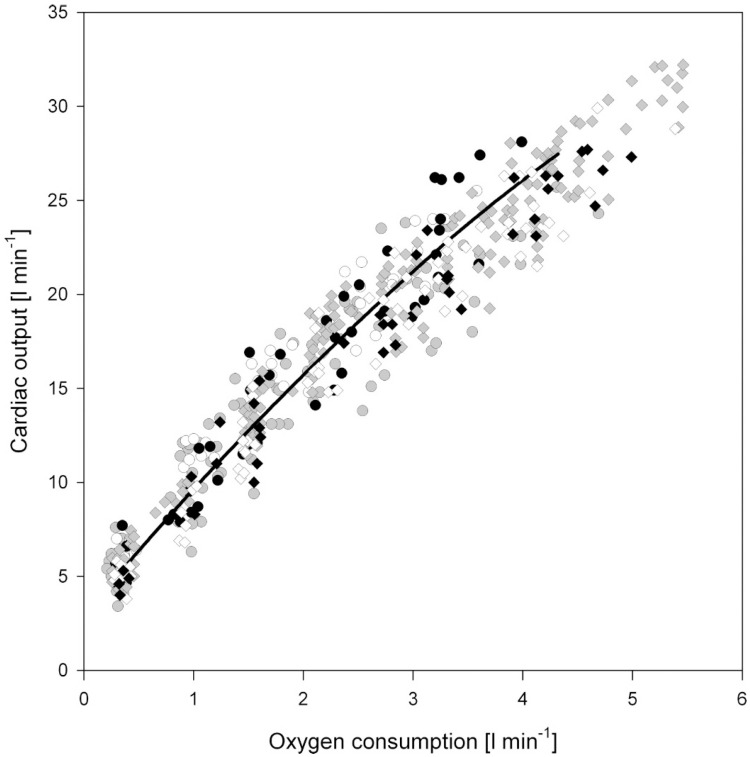
Simulated and experimental relationship between cardiac output and $\dot{V}$O_2_. Taken with permission from Liguzinski and Korzeniewski [12].

**Figure 4:**
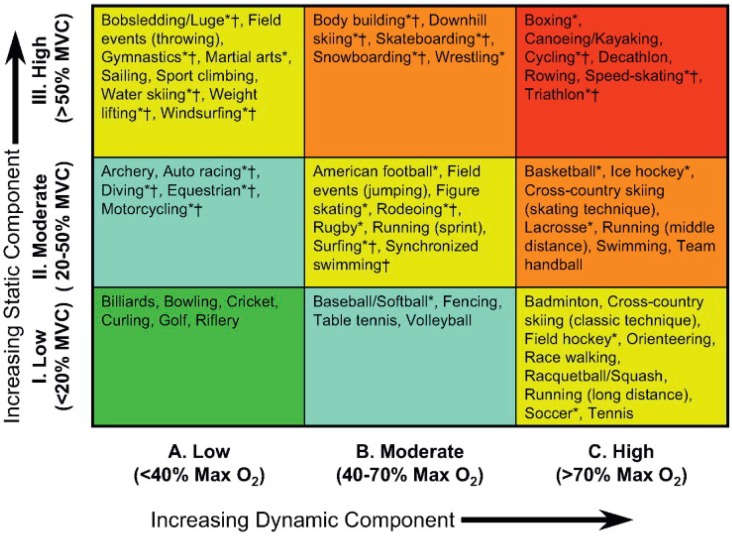
Sports classification model highlighting the combination of static and dynamic characteristics of differing sporting activities. Taken with permission from Mitchell et al. [13].

Aerobic exercise is associated with an increased metabolic and thermoregulatory demand. For exercise to continue for any period of time beyond a few seconds, these competing demands must be met by an enhanced delivery of blood, both to the working muscle and the capillary beds responsible for heat exchange. This unavoidable need for blood flow to increase during exercise, explains the close relationship between cardiac output ($\dot{Q}$*—the product of heart rate and stroke volume*) and the volume of oxygen consumed per minute ($\dot{V}$O_2_) (See Figure 3) [12]. In humans, during dynamic (isotonic) aerobic exercise, heart rate and stroke volume increase, alongside a modest reduction in overall systemic vascular resistance; the net result being a large increase in cardiac output. In contrast, during resistance or static (isometric) exercise, a more modest increase in cardiac output is observed that is predominantly driven by an increase in HR, but which is also accompanied by a more pronounced rise in systemic blood pressure than observed during dynamic aerobic exercise. Hence, the hemodynamic load imposed on the heart is dependent upon the exercise intensity, duration, and modality. Importantly, it should be noted that exercise is very rarely either dynamic or static in isolation, rather, the majority of sports fall somewhere along a continuum that involves both elements. In an attempt to better characterize the relative load of individual sports, Mitchell and colleagues [13] have proposed an extended matrix (Figure 4), which provides a far better representation of the “mixed” cardiovascular load that athletes hearts are exposed to. Despite this, much of the available literature regarding cardiac remodeling in human athletes due to exercise has been presented within the context of either an exercise-induced pressure or volume stimulus.

### 1.2. Athlete’s Heart: Evidence from Humans

Cardiac remodeling in response to athletic training is a well-described phenomenon in humans [14,15]. As early as the 1890s, using auscultation and percussion, physicians in Sweden and the USA demonstrated increased cardiac dimensions in elite cross-country skiers [16] and rowers, respectively [17]. These data were followed by the first reports of athletic bradycardia in runners from the Boston Marathon [18]. Subsequently, with improved imaging technologies (e.g., cardiac ultrasound and magnetic resonance imaging), many studies have gone on to characterize the structural and functional adaptations observed in the human heart in response to athletic training. Since the mid-1970s, the field was driven by a dichotomous characterization of the athlete’s heart. Specifically, it has been suggested that endurance athletes present with cardiac adaptations, induced by an increased “volume” load, while power-based athletes have a cardiac phenotype shaped by a higher “pressure” load. Simplistically, endurance athletes have large eccentrically remodeled hearts—*large ventricular volumes*, *modest wall thickening, and a low relative wall thickness*—associated with reductions in resting heart rate, while power athletes present with concentric remodeling—*thick ventricular walls*, *relatively small ventricular volumes*, *and a high relative wall thickness with minimal change in heart rate.* The latter is postulated to be induced by spikes in systemic blood pressure, and hence ventricular wall stress associated with repetitious strength/power-based activities. This dichotomous differentiation between endurance and power athletes was initially made by Morganroth [19], and has subsequently been termed the “Morganroth hypothesis”. Until recently, this view has been widely accepted in the sports cardiology literature. However, whilst this hypothesis is attractive from a physiological basis, it is likely an over-simplistic representation of cardiac remodeling in response to athletic training.

The data for the original hypothesis was derived from early studies, using limited one-dimensional imaging techniques [19]. As imaging modalities have advanced, authors have theoretically [20] and empirically [21] argued against concentric remodeling in response to resistance-based exercise training. These authors cite inadequate approaches to cardiac scaling, insensitive imaging modalities, and the potential influence of anabolic agents in some of the populations studied, as reasons why early work supported concentric hypertrophy in these athletes. Haykowsky and colleagues [22] have provided further physiologic rationale as to why resistance athletes may not experience concentric remodeling. Individuals completing intense resistance training (e.g., power lifting) often perform a Valsalva manoeuvre during individual efforts. This respiratory manoeuver equalizes transmural pressures and so the wall stress, or afterload, faced by the left ventricle (LV), is not significantly greater than at rest [22]. Thus, if athletes do perform a Valsalva during resistance activities, the heart is not exposed to the adaptive stimulus. Although Haykowsky’s data are compelling, it should be noted that not all resistance activities are performed with a Valsalva manoeuvre. Often, the nature of the athletic activity precludes its use, particularly when the activity is dynamic in nature. Accordingly, it is likely that in all but the most short-lived controlled resistance activities, athletes will experience repeated increases in cardiac afterload, to varying extents.

Presenting longitudinal data from American college football athletes, Weiner and colleagues [23] confirmed the presence of concentric remodeling in response to a prolonged period of resistance-based training. It should be noted, however, that these athletes also developed modest hypertension alongside the athletic training. As such, the increased LV afterload was not confined to the periods of training, and the observed increase in ventricular wall thickness cannot be solely attributed to the athletic stimulus. In contrast, Spence and colleagues [21], using cardiac MRI images, failed to show concentric remodeling in response to six months of resistance exercise training. It should be noted, however, that these individuals were naïve to training before the start of the study, and the training stimulus was limited to three one-hour sessions per week. Therefore, these results cannot be directly compared to the literature derived from competitive athletes who complete a far greater volume of training, and have done so for a considerably longer period of time.

A number of authors have adopted meta-analytical techniques for exploring the Morganroth hypothesis. The most recent of which confirmed evidence of eccentric remodeling in endurance-trained athletes [24], but also provided some support for cardiac remodeling in resistance-trained athletes. Although there were markedly fewer resistance-training studies included in the analyses, both the inter-ventricular septal wall and posterior wall thicknesses were greater in resistance-trained athletes than controls, and were similar to the values seen in endurance athletes. Ventricular cavity dimensions, but not volumes, were also greater in resistance athletes than the controls, but were lower than in the endurance athletes. In a previous meta-analysis, Pluim and colleagues [25] also provided support for concentric hypertrophy in resistance-trained athletes, based on an increased relative wall thickness (RWT, ratio of wall to chamber dimension). However, mean RWT data from Utomi et al. [24] were 0.40 for endurance athletes and 0.41 in resistance athletes, both within clinical norms and not significantly different. The authors therefore suggested that, “*as opposed to dichotomous cardiac structural responses to endurance and resistance training*, *it might be argued that both athlete groups present with a similar qualitative cardiac adaptation on a continuum*, *with greater cardiac dimensions in endurance athletes reflecting a greater overall training volume*”. Fundamental to the Morganroth hypothesis is the assumption of a dichotomous hemodynamic load in sports; that is endurance and resistance athletes exclusively experience a volume or pressure overload, respectively. As outlined above, this is not the case for the vast majority of athletes; most athletes engage in a combination of resistance and endurance activities, and thus experience a combination of static and dynamic cardiovascular loads [13]. In all likelihood, as suggested by Utomi and colleagues [24], a continuum exists where the LV adapts in response to the many and varied acute and chronic hemodynamic challenges, induced by exercise training.

### 1.3. Cardiac Adaptations to Exercise Training beyond the Left Ventricle

To date, the vast majority of work examining the athlete’s heart has focused on the LV. This is likely a consequence of the important role the LV plays in generating the cardiac output required to meet the demands of exercise, but is also probably related to the relative ease associated with imaging the left side of the heart. Notwithstanding, an increasing body of work has been completed which examines the potential of the right ventricle, atria, and aorta to remodel, in response to training in human athletes.

**Figure 5:**
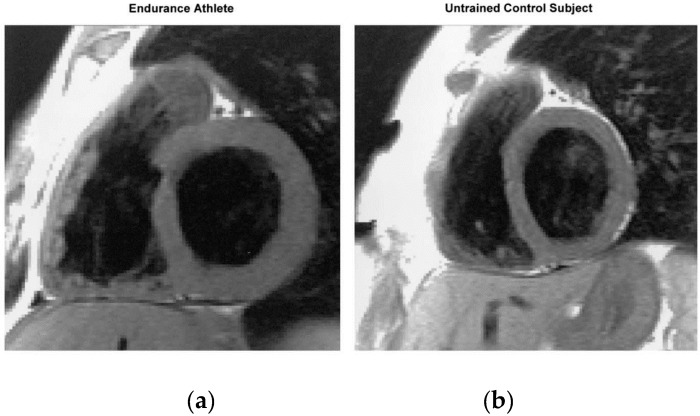
End-diastolic T1-weighted short-axis slice from an endurance athlete (**a**) and an untrained control subject (**b**). Compared with the heart of the control subject, the endurance athlete’s heart is characterized by an enlarged volume and a greater myocardial mass of both ventricles, while the proportions of the left and right heart are the same as in the untrained control subject. Taken with permission from Scharhag et al. [26].

Given that exercise requires the stroke volume to increase in both the left and right ventricles, and that there is clear evidence of LV remodeling in endurance athletes, it should not be surprising that the RV also remodels. If there was not a proportionate increase in RV size, then a non-sustainable mismatch would occur in what is largely a closed-loop system. Using MRI images, Scharhag and colleagues [26] have provided evidence of the balanced bi-ventricular remodeling in elite endurance athletes (Figure 5). Although, in absolute terms, the resting afterload faced by the RV is considerably less than the LV, the rise in pulmonary artery pressure with exercise is proportionally greater than the rise in systemic arterial pressure [27]. This increase in RV afterload is related to both the inelastic properties of the pulmonary artery, and a relative lack of vasodilation in the pulmonary circulation. Accordingly, similar to the LV, there is a considerable stimulus for RV enlargement in athletes completing large volumes of endurance training. At present, less is known regarding RV remodeling in resistance-trained athletes and this requires further study [24].

Once again, drawing on the results from meta-analyses, it is apparent that the aorta [28] and left atrium respond to athletic training [29]. Using data from 23 previous studies, Iskandar and colleagues [28] showed that the aortic root is on average 3.2 mm lager, and the aortic valve annulus 1.6 mm greater, in athletes when compared to controls. The difference, however, appeared more marked in endurance-, than resistance-trained, athletes. The authors rightly recognize that they cannot rule out the potential confounding effects of body size on their findings, as larger individuals would unsurprisingly have larger aortas. In a similar fashion, the same authors [29] pooled data from 54 studies, including more than seven thousand athletes (endurance, strength, and combined athletes), in order to examine athletic remodeling of the left atria. Their analysis showed that the weighted mean left atrial diameter was 4.1 mm greater in athletes, in comparison to controls, and that LA volume indexed to body surface area was 7 mL/m^2^ greater in athletes. Similar to the LV and the aorta, endurance athletes appear to show the greatest adaptation in the LA, but there is also some evidence of remodeling in resistance- and combination-trained athletes.

### 1.4. Cardiac Mechanics

Recently, emerging technology has enabled the assessment of the mechanics that underpin LV function (e.g., deformation of the myocardium in the longitudinal, radial, and circumferential planes, and counter-directional rotation of the ventricular base and apex). Cardiac mechanics are intimately connected to the underlying myocardial architecture, which as outlined above, remodel significantly in response to exercise training. Authors have therefore sought to examine the effect of exercise training on LV mechanics. This is an emerging field, yet early data suggest that highly fit individuals have lower resting mechanics [30,31]. Similar to the bradycardia induced by endurance training, it is likely that a lower degree of myocardial deformation at rest facilitates a greater reserve to draw upon during exercise. Such a reserve would aid diastolic filling, and augment SV and cardiac output during periods of increased demand. Recent work has shown that this adaptation in ventricular mechanics is likely to be dependent upon the duration of training experience. During the initial phase of athletic training, LV rotational mechanics appear to increase, possibly related to an enhanced blood volume, which is then followed by a normalization, or even reduction, in the mechanics once the myocardium itself has remodeled [32].

## 2. Summary

Since the 1800’s, a significant body of work has been completed showing marked cardiac adaptations in the human heart in response to athletic training. These adaptations appear to be modulated by the relative volume and pressure load faced by the heart. This, in turn, is modulated by the type of activity undertaken by the athletes, which cannot be equated as simply either pressure or volume. As imaging modalities continue to improve, it is likely that we will gain a better understanding of the relationship between cardiac structure, mechanics, and overall function. Importantly, as this field advances, it will be important to move away from the pervasive dichotomous contextualisation of exercise training, to increase the number and quality of longitudinal studies, and to attempt to examine the influence of cardiac remodeling on cardiac function during both endurance and resistance exercise.

### 2.1. Cardiac Remodelling in Non-Human Mammalian Athletes

Many mammals are capable of impressive athletic performance, underpinned by highly adapted physiology [3]. Although the top speed (~70 mph) achieved by the cheetah [33] is extremely impressive, the combination of speed and endurance demonstrated by the pronghorn antelope (50 mph for several miles) [3], or the African Wild dog (41 mph for 10–60 min) [34], is equally, if not more impressive, given the physiology required to sustain such activity. These wild animals have evolved to fill a specific niche; however, through domestication, selective breeding, and tailored training practices, humans have artificially selected animal breeds capable of exceptional athletic performance. For example the Quarter Horse reaches maximal speeds of ~57 mph [35] and the Alaskan sled dog, during the Iditarod 1000+, can sustain a $\dot{V}$O_2_ in excess of 100 mL/kg/min, for more than 10 h a day [3]. Such performance is underpinned by a highly adapted physiology and the ability to support both the metabolic and thermoregulatory demands of such activity. As discussed in the introduction above, the cardiovascular system is intimately linked to athletic performance, and it therefore follows that the heart of horses and dogs bred, and trained, specifically for performance, will have adapted and physiologically remodelled to meet the specific demands of the exercise imposed upon them.

### 2.2. The Heart of Athletic Horses

The level of endurance performance commonly observed in horses is only possible with a highly efficient physiology, primed to take in and transport large volumes of oxygen required for aerobic metabolism. The heart rate range of the horse is large, from ~26 b·min^−1^ at rest, to ~240 b·min^−1^ during intense exercise. This large HR reserve is coupled with the ability to double the packed-cell volume through splenic contraction [36]. When considered as part of the Fick equation:

$$\dot{V}O_{2max}\;=\;maximal\;cardiac\;output\;\times \;maximal\;a-VO_{2difference}$$

these adaptations are of huge benefit in optimising and aiding oxygen transport and extraction, respectively. Although the maximum heart rate is important in the determination of maximal cardiac output, it does not appear to differ between age-matched horses, and is not affected by exercise training [37]. As such, stroke volume in the equine athlete is probably the biggest determinant of $\dot{V}$O_2max_.

**Figure 6:**
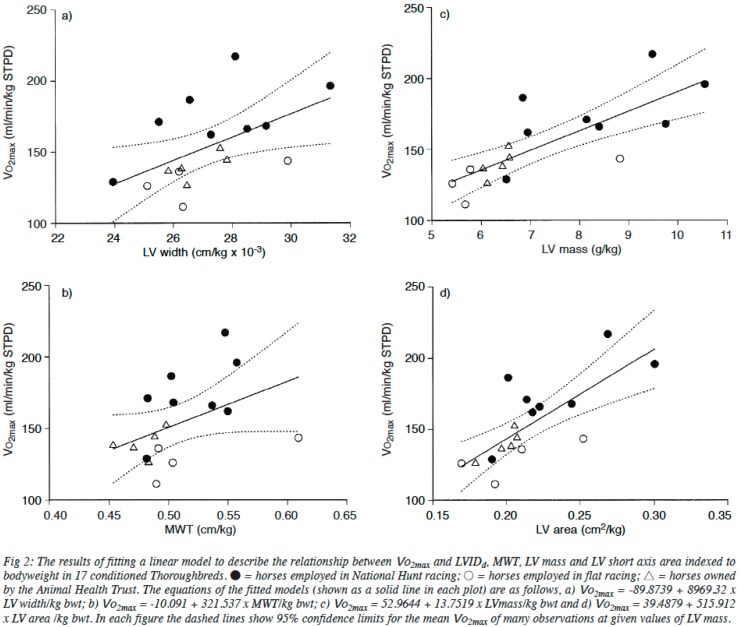
Relationships between various measures of heart size (*LV width* (***a***), *mean wall thickness* (***b***), *LV mass* (***c***), *LV area* (***d***)) and $\dot{V}$O_2max_ in a range of horses. Taken with permission from Young et al. [38].

Similar to the early work in human athletes, reports of large hearts in equine athletes date back several hundred years. Following the death of Eclipse in 1789, a horse that was unbeaten over 26 races, his heart was extracted and reportedly weighed, with a measurement of ~6.3 kg [3]. Similarly, at autopsy, the heart of the highly successful American racehorse Secretariat, was estimated to be ~10 kg. In comparison to normal horses, whose hearts weigh ~3.9 kg, hearts of these sizes would be capable of generating extremely large cardiac outputs. Examining the link between heart size and aerobic capacity in horses, Young and colleagues [38] have shown strong positive relationships between various measures of heart size and $\dot{V}$O_2max_, a key determinant of endurance performance (see Figure 6). Based on the heart mass of Secretariat, it has been estimated that his maximal cardiac output would have been in excess of 500 L·min^−1^, which could have supported a $\dot{V}$O_2max_ of approximately 240 mL·kg·min^−1^ [3]. Unsurprisingly, there is also a relationship between race performance and heart size; Young et al. [39] have demonstrated a significant linear correlation between the British Horseracing Board Official rating or Timeform rating in National Hunt racing, and heart size, as assessed by ultrasound. This relationship has subsequently been confirmed in other horses, but specifically in horses where performance is dependent upon aerobic capacity, and thus cardiac output [40].

Although previous cross-sectional work in horses has shown that heart size is related to athletic performance, this does not prove a “cause and effect” relationship between athletic training and cardiac remodeling. Despite this limitation, there is evidence to support adaptive cardiac remodeling in response to exercise training, much the same as that previously described in humans. For example, autopsy studies have confirmed that trained Thoroughbreds have larger hearts than their untrained counterparts [41]. This has been further supported by longitudinal echocardiographic studies showing differences in cardiac structure between horses of differing training status [42,43]. Buhl and colleagues [43] examined the impact of approximately 18 months of exercise-training on cardiac structure and function, in 103 Standardbred horses. From the start to the end of the training period, internal ventricular diameters in diastole, LV mass, and mean ventricular wall thicknesses, all increased. Relative wall thickness, however, did not, suggesting that the Standardbred horse’s ventricle undergoes eccentric remodeling, similar to that observed in human endurance runners. This contrasts with data from a previous longitudinal study examining Thoroughbred horses [42], which in addition to the increases in cardiac dimensions, documented an increase in RWT from 0.40 to 0.45, suggesting that these equine athletes, in comparison to the Standardbreds, experience a mixed ventricular remodeling. It is possible that Thoroughbred horses undergo a more demanding training regime, or that they experience greater elevations in systemic pressures during exercise, than Standardbreds. Of note, while mean systemic pressures in elite horses have been documented in excess of 200 mmHg [3], it has been shown that Thoroughbreds experience higher pulmonary capillary and wedge pressures, than Standardbreds at similar relative exercise intensities [44], and so it is entirely possible that the exercise-related hemodynamics on the left side of the heart are also markedly different.

Similar to human endurance athletes, right ventricular adaptations have also been observed in Thoroughbreds, following intense training. Lightfoot and colleagues [45] showed increases in right ventricular dimensions following a period of intense physical training. Such remodeling is likely related to the volume load experienced by the right side of the heart, induced by frequent elevations in venous return, and increases in total blood volume [46]. However, due to the difficulties in imaging the right ventricular free wall, the authors were unable to assess whether RV wall thickness also increased. Given the marked elevations in pulmonary artery pressures, and the increased blood viscosity related to the transient elevations in packed cell volume previously noted, it is likely that RV wall thickness would also increase, in order to minimize wall stress.

A finding repeatedly observed in the equine literature is that of an increased incidence of valvular incompetence in highly trained horses. The most common cardiac murmur is tricuspid valve regurgitation [47,48], the prevalence and severity of which is associated with both age and years of training [49]. In a prospective study completed by Lightfoot and colleagues, a simultaneous increase in RV diameters was concurrently observed with an increased incidence of tricuspid regurgitation, assessed using color flow Doppler (ranging from 10% of horses before training, to 23% after the initial 8–12 weeks of training, to 36% of all animals at full race fitness). Although it is impossible to suggest cause and effect, it appears likely that the RV remodeling experienced by the horses, alters the ventricular hemodynamics and hence propensity for regurgitation in this population of Thoroughbreds.

In summary, it is clear that the horse’s heart is well adapted for endurance exercise and appears plastic in response to intense athletic training. Importantly though, the quadrupedal nature of locomotion, the coupling of respiration to stride frequency, the propensity to markedly increase oxygen carrying capacity through splenic constriction, and the pronounced increases in pulmonary artery pressure, all mean that the physiologic load placed upon the heart during exercise is very different to that observed in humans. As such, whilst we can learn a great deal from the horse in relation to athletic cardiac remodeling, it is very likely that species-specific phenotypic adaptations are present and responses to training may well differ. Future work using advanced imaging techniques, is required to develop a better understanding of the athletic heart syndrome in horses. Such work is warranted in order to better understand the physiology of equine athletes, but more importantly, to help address the causes of exercise-associated sudden cardiac death, which have been associated with the extreme physiology of horseracing [50,51].

### 2.3. The Athletic Heart in Dogs

In the wild, canids hunt for prolonged periods of time, and as such, are well adapted to meet the demands of aerobic exercise. Since their domestication at least 15,000 years ago [52], dogs have undergone artificial selection, resulting in the development of phenotypic traits suited for specific activities (e.g., terriers for digging, spaniels and retrievers for hunting and retrieving games, and sheep and cattle dogs for herd and flock management). It is likely, therefore, that through this process, the cardiovascular system of dogs has been directly or indirectly selected to facilitate the activities that the animals have been bred for. Surprisingly, however, relatively little work has been completed examining the cardiovascular structure and function in different breeds of dogs, and even less has been completed that considers the acute (within animal) or chronic (across generations) effect of exercise training. For example, generic echocardiographic guidelines for “dogs” are still often used when assessing cardiac structure and function for pathology [53,54]. If athletic remodeling does occur in dogs, the use of such generic guidelines could result in clinical confusion and inappropriate diagnosis [55].

Although relatively little work exists explicitly examining the effects of exercise on the canine heart, a small number of informative studies have been completed and provide the basis for our current understanding of exercise-induced cardiac remodeling in dogs. Similar to athletic humans, it has been shown that athletic training results in a reduction in resting HR in dogs [56,57,58,59,60]. Furthermore, the heart rate response to a standardized atropine challenge [57] or a fixed exercise workload [61,62], is also reduced following periods of exercise training. These lower resting, and sub-maximal heart rates, coupled with a maximum HR of ~300 b·min^−1^ [63], provide a large cardiac reserve in trained dogs that would help to meet the metabolic and thermoregulatory demands of exercise.

In contrast to the HR response to exercise training, there has been some conflict regarding the potential of structural cardiac remodeling in dogs. In early studies, increases in wall thickness and LV mass were consistently reported in response to exercise training [61,64], however, whether internal LV dimensions or volumes increased, was debated [61]. These early discrepancies likely arose due to the wide range of methodologies adopted, and importantly the different breeds of dogs studied. Notwithstanding, from a comparative perspective, it would be extremely interesting and informative if dogs do not remodel in a similar fashion to humans, in response to a similar endurance exercise stimulus.

Given the athletic prowess of the Greyhound, it is not surprising that this breed has featured heavily in the studies that have been completed examining the canine heart. Greyhounds are capable of running at almost 50 mph, and a single [65] animal has been recorded with a $\dot{V}$O_2max_ of approximately 270 mL·kg·min^−1^ [3]. In comparison to breeds of a similar size, greyhounds have larger internal LV diameters, LV wall thicknesses, and higher heart to body weight ratios [65,66,67]. Similar findings have recently been confirmed in smaller racing dogs, such as Salukis and Whippets [68].

Previously, it has been debated whether the larger hearts in greyhounds are determined genetically, or reflect adaptations to training [67,69]. However, when training greyhounds were compared to non-training animals, their ventricular diameters, wall thicknesses, and cardiac output were shown to be larger [69,70], suggesting at least some degree of athletic-induced remodeling. Such remodeling of the ventricle should not be unexpected, especially as mean arterial blood pressure in the trained greyhound has been shown to be significantly higher than in mongrel dogs, or untrained greyhounds [69,71]. Similarly, athletic training has also been shown to result in an increased blood volume [59,72], which likely acts as a stimulus for remodeling of the ventricular cavity. Evidence therefore suggests that the large heart of the greyhound is genetically conferred, but it seems likely that athletic remodeling in response to both a pressure and volume load, also influences its cardiac morphology.

Another breed of dog capable of prodigious endurance performance is the Alaskan sled dog [73,74]. In the most comprehensive study examining exercise-induced cardiac remodeling in dogs, Stepien and colleagues [60] longitudinally assessed cardiac structure and function in 77 Alaskan sled dogs, before and after five months of endurance training. The training consisted of running ~20 km each day, pulling a sled and musher which was estimated to equate to ~30%–40% $\dot{V}$O_2max_. Training was associated with significant increases in ventricular wall thicknesses (9%) and cavity dimensions (4%). The study included both veteran (*having completed at least one previous season of training*) and rookie (*in their first season*) dogs, and although ventricular wall thickness increased in both, following training, the remodeling was greater in the rookies. This finding likely relates to both the difference in biological age between the two groups, and also the retention of cardiac hypertrophy in the veteran dogs since the previous season.

The proportionally greater increase in ventricular wall thickness observed in the sled dogs following training, resulted in an increased relative wall thickness, which the authors suggest was attributable to a combined volume and pressure load associated with sled pulling exercise. While there is undoubtedly a brief period of resistance exercise associated with overcoming the initial inertia of the sled and musher, once moving, it would seem unlikely that the exercise undertaken is truly “resistive” in nature. As the authors themselves suggest, the exercise intensity equated to approximately 30%–40% $\dot{V}$O_2max_. We therefore question whether the mixed ventricular remodeling observed is due to the “pressure load” induced by pulling the sled. Clearly, for wall thickness to increase to a greater extent than the internal cavity, a pressure load on the heart is required. However, instead of the exercise itself being resistance in nature, it is possible that the exercise *per se* is associated with a large pressure load in these animals. In chronically instrumented sled dogs, Van Citters and Franklin showed that systolic pressures could exceed 300 mmHg during aerobic exercise [75]. These pressures far exceed those seen in humans undertaking endurance exercise [76]. Accordingly, the pressure load described by Morganroth [19], to be associated with resistance exercise in humans, may be obligatory in sled dogs during endurance activity. This may therefore explain the increases in absolute and relative ventricular wall thickness, observed by Stepien and colleagues. Further work is needed to understand the blood pressure response to exercise in dogs, especially across different breeds. However, it is possible that the high systolic pressures observed in the sled dogs may be related to quadrupedal hemodynamics, or to differences in thermoregulation between dogs and humans (e.g., panting vs. sweating). These potential differences in canine hemodynamics may help to resolve why increases in ventricular wall thicknesses are a consistent finding in athletic dogs capable of significant endurance performance.

To summarize, while some work has been completed examining cardiac adaptation to exercise training in a small number of dog breeds, in general, there is a paucity of data regarding athletic remodeling in the canine world. As dogs have been bred for a variety of different tasks, it is highly likely that the cardiac phenotype will differ between breeds and how this phenotype responds to different training stimuli, will also possibly vary. Until more work is completed, when examining different breeds and different training interventions, care should be taken when trying to extrapolate findings from one breed to another.

## 3. Conclusions

In contrast to the human field, cardiac remodeling induced by athletic training has only received limited attention in the veterinary literature. As such, the subtleties of cardiac structure and function, which have been examined in great detail in human athletes, are yet to be fully discerned in our equine or canine equivalents. While clear data do exist regarding cardiac remodeling in human athletes, it should be noted that the response to training is heterogeneous. It is likely that this heterogeneous response is partially explained by differences in exercise stimulus, but genetic and epigenetic factors are also likely involved [77]. In contrast, the artificial selection imposed by man on both horses and dogs for athletic performance, likely means that these athletes are far more genetically similar than their human counterparts. Therefore, the study of cardiac remodeling in response to athletic training in these animals may provide greater insight into the potential for athletic activity to remodel the heart, independent of confounding variables. It should, however, be remembered that any adaptation observed in response to athletic training is superimposed on a species-specific, or indeed breed-specific, phenotype, which has been shaped by evolution and/or selective breeding. Care should therefore be taken when inferring between species or breeds in relation to exercise-induced cardiac remodeling.
